# Uncovering the stability status of the reputed reference genes in breast and hepatic cancer cell lines

**DOI:** 10.1371/journal.pone.0259669

**Published:** 2021-11-09

**Authors:** Gilar Gorji-Bahri, Niloofar Moradtabrizi, Atieh Hashemi

**Affiliations:** Department of Pharmaceutical Biotechnology, School of Pharmacy, Shahid Beheshti University of Medical Sciences, Tehran, Iran; East Carolina University, UNITED STATES

## Abstract

Accurate and reliable relative gene expression analysis via the Reverse Transcription-quantitative Real Time PCR (RT-qPCR) method strongly depends on employing several stable reference genes as normalizers. Utilization of the reference genes without analyzing their expression stability under each experimental condition causes RT-qPCR analysis error as well as false output. Similar to cancerous tissues, cancer cell lines also exhibit various gene expression profiles. It is crucial to recognize stable reference genes for well-known cancer cell lines to minimize RT-qPCR analysis error. In this study, we showed the expression level and investigated the expression stability of eight common reference genes that are *ACTB*, *YWHAZ*, *HPRT1*, *RNA18S*, *TBP*, *GAPDH*, *UBC*, and *B2M*, in two sets of cancerous cell lines. One set contains MCF7, SKBR3, and MDA-MB231 as breast cancer cell lines. Another set includes three hepatic cancer cell lines, including Huh7, HepG2, and PLC-PRF5. Three excel-based softwares comprising geNorm, BestKeeper, and NormFinder, and an online tool, namely RefFinder were used for stability analysis. Although all four algorithms did not show the same stability ranking of nominee genes, the overall results showed *B2M* and *ACTB* as the least stable reference genes for the studied breast cancer cell lines. While *TBP* had the lowest expression stability in the three hepatic cancer cell lines. Moreover, *YWHAZ*, *UBC*, and *GAPDH* showed the highest stability in breast cancer cell lines. Besides that, a panel of five nominees, including *ACTB*, *HPRT1*, *UBC*, *YWHAZ*, and *B2M* showed higher stability than others in hepatic cancer cell lines. We believe that our results would help researchers to find and to select the best combination of the reference genes for their own experiments involving the studied breast and hepatic cancer cell lines. To further analyze the reference genes stability for each experimental condition, we suggest researchers to consider the provided stability ranking emphasizing the unstable reference genes.

## Introduction

The last statistics of WHO have shown that breast and liver cancers are among the six most prevalent cancers in both sexes. Breast cancer is accounted for the deadliest cancer in women. Whilst in men, after lung cancer, liver cancer is listed as the most lethal cancer all around the world [1]. Owing to the high prevalence of cancer in the whole world, studies on gene expression fluctuations both for therapeutic and diagnostic purposes have received much attention from researchers [2, 3]. One of the most useful, sensitive, and rapid techniques to analyze and compare genes expression levels is relative gene expression analysis via RT-qPCR, in which the mRNA expression level of a target gene is normalized against the expression of one or more reference genes [4]. The reliability and accuracy of the results directly depend on the choice of the appropriate reference genes. A reference gene with the same expression level in samples subjected to various treatments or in different physiologic and pathologic conditions is considered as a stable reference gene [5, 6]. Moreover, to maximize the accuracy of RT-qPCR data normalization, it is suggested to select reference genes with a close expression level to the target gene [6]. However, stability analysis of the reference genes previously showed that various tissue types might express different levels of a specific reference gene [6, 7]. Therefore, it is critical to evaluate and discern stable reference genes for each tissue type separately to have reliable outcomes of the RT-qPCR method.

The utilization of various established cancer cell lines instead of the primary cells facilitates studying the molecular mechanisms of cancers. According to the primary source of immortalized cancer cell lines, each line would reflect the characteristics of a specific classification of a type of cancer and has a distinct gene expression profile. For example, breast cancer cell lines such as MCF7, MDA-MB-231, and SKBR3 fairly represent different classifications of this heterogeneous cancer. Among them, MCF7 and MDA-MB231 cell lines are both human epidermal growth factor receptor 2 (HER2) negative, while MDA-MB231 is estrogen receptor (ER) negative too. In contrast to MCF7, SKBR3 is HER2 positive and ER-negative [8]. Besides that, HepG2, a well-known hepatocellular carcinoma cell line, was extensively considered for genotoxicity studies [9]. Moreover, varied gene expression profiles have been reported for four hepatic cancer cell lines, namely Huh7, HepG2, Hep3B, and Huh6 [10].

Accordingly, relative gene expression analysis in cell lines of a specific type of cancer strongly requires knowledge about the stable reference genes in the studied cell lines. Many researches have determined stable reference genes in cell lines under different treatment or conditions, such as silver nanoparticles [11] and hypoxia [12], respectively. Based on our searches, we did not find a study that examined the reference genes’ stability in several cancer cell lines of a type of cancer. However, frequently utilized reference genes like glyceraldehyde-3-phosphate dehydrogenase (*GAPDH*), regardless of their stability analysis, have been repeatedly reported in the literature [13]. Thus, in this study, the stability of eight useful reference genes in two sets of cancer cell lines, including hepatic cancer cell lines (HepG2, PLC-PRF5, and Huh7) and breast cancer cell lines (MCF7, MDA-MB231, and SKBR3) was analyzed via four distinct algorithms (GeNorm, NormFinder, BestKeeper, and RefFinder), which allows us to compare the expression and stability ranking orders of reference genes between two types of cancer. Moreover, the stability ranking of these reference genes in both hepatic and breast cancer cell lines in a simultaneous manner is also reported. Undoubtedly, considering the results of our study will accelerate and facilitate the selection of suitable reference genes in studies focusing on hepatic and breast cancer cell lines, which provide reliable RT-qPCR analysis.

## Materials and methods

### Cell culture

Hepatic cancer cell lines including HepG2, Huh7, and PLC-PRF5, and breast cancer cell lines including MDA-MB231, MCF7, and SKBR3 were obtained and cultured as follows: HepG2, PLC-PRF5, MDA-MB231, MCF7, and SKBR3 were bought from Pasteur Institute of Iran and cultured in RPMI 1640 medium (Inoclon, Iran). Huh7 cell line was purchased from Royan Institute of Iran and cultured in DMEM high glucose medium (Inoclon, Iran). The medium of all six cell lines was supplemented with 10% FBS (Gibco, USA) and 1% penicillin/streptomycin (Biosera, France). Cells were kept up in a humidified incubator with 37 Ċ temperature and 5% CO2.

### Selection of reference genes, designing the primer pairs, and calculation of PCR efficiency

Reference genes and corresponding primers used in this study were the same selected and designed in our previously published articles [11, 14–16]. Evidences considering the usage of these reference genes in hepatic and breast cancer cell lines are presented in Table 1; not exactly the cell lines investigated in this study. The specificity of primer pairs was checked via 1% agarose gel electrophoresis and melt curve analysis. The primer pairs spanned at least one intron or exon-exon junction, resulting in amplification of mRNA rather than genomic DNA.

**Table 1:** Properties of the designed primers of the eight nominee reference genes.

Gene	Accession number	Mean PCR efficiency ± SD	Evidences
*GAPDH*	NM_002046.6	1.903 ± 0.026	[2, 13, 18–21]
*B2M*	NM_004048.4	1.913 ± 0.009	[4, 22]
*UBC*	NM_021009	1.908 ± 0.023	[23]
*YWHAZ*	NM_003406	1.903 ± 0.019	[23]
*ACTB*	NM_001101.5	1.917 ± 0.022	[2, 21, 24, 25]
*RNA18S*	NR_003286	1.935 ± 0.025	[26–29]
*TBP*	NM_003194	1.908 ± 0.022	[23]
*HPRT1*	NM_000194.3	2 ± 0.024	[4, 30]

The Mean PCR efficiency ± SD refers to the average of PCR efficiencies of each reference gene across all six cell lines and all biological and technical replicates.

PCR efficiency (E) was also provided using LinregPCR version 2017 software. Instead of making serial dilutions and drawing standard curves which may cause errors in PCR efficiency calculation due to common pipetting errors, the LinregPCR software calculates PCR efficiency for individual reaction via calculating the slope of the regression line in an exponential phase of the amplification curve, namely Window-of-Linearity (W-o-L) area, and reports the mean PCR efficiency of samples containing same primer pairs [14, 17]. Using the LinregPCR software, the imported raw amplification data of each RT-qPCR run, belong to each reference gene analysis was automatically corrected for baseline adjustment, and at least 3 points were incorporated for the W-o-L area of each sample. Finally, the mean PCR efficiencies were reported as a value between 1 and 2, in which the 2 value refers to 100% amplification efficiency. In comparison, the 1 value refers to no amplification. Only the mean PCR efficiency ± SD values in Table 1 are specific for this study. Sequences of primer pairs were already published by our team [11, 14–16].

### Isolation of total RNA and synthesis of complementary DNA (cDNA)

Total RNA of each cell line was isolated individually using Trizol reagent (Invitrogen, USA) according to the manufacturer’s instruction with some modifications. Briefly, 80% confluent T25 flask, containing approximately 3 × 10^6^ cells were lysed via 1 ml Trizol reagent and incubated for 5 min at room temperature. Subsequently, 200 μl chloroform was added to the sample, shook vigorously for 15 sec, and incubated for 15 min at room temperature. After that, phase separation was performed via centrifugation at 12000 ×g for 15 min at 4 Ċ. The clear aqueous phase supernatant containing RNA was carefully separated from genomic DNA at the interphase, using 10 μl pipette tips. To precipitate RNA, 500 μl cold isopropanol was added to the sample and incubated for 10 min at -20 Ċ, followed by centrifugation at 12000 ×g for 10 min at 4 Ċ. The precipitated RNA was suspended in 1 ml 75% ethanol and centrifuged at 7500 ×g for 5 min at 4 Ċ. The RNA pellet was air-dried at room temperature and finally dissolved in 30 μl pre-warm (55 Ċ) ultrapure DEPC-treated water (Invitrogen, USA). Using 1% agarose gel electrophoresis, *RNA28S*/*RNA18S* band intensity ratio was checked, and the integrity of the extracted RNA was confirmed. The 260/280 absorbance ratio was also checked to confirm the purity of the isolated RNA (HTX multimode reader (BioTek)). Finally, the high-quality isolated RNA was quantified, stored at about -80 Ċ until use within six months.

Using cDNA synthesis kit (Yekta Tajhiz, Iran), cDNA synthesis was carried out as stated in the manufacturer’s instruction, in which total RNA of each cell line (1 μg), random hexamer primer (50 μM), and MMLV reverse transcriptase (200U/μl) along with dNTPs (10 mM), 5X first strand buffer and RNAse inhibitor (40 U/μl) were included in 20 μl volume reaction. The synthetized cDNA was stored at about -20 Ċ until use within two weeks.

### RT-qPCR

Ten microliter volume reactions containing 5 μl SYBR premix Ex TaqII (2X) (Takara, Japan), 1 μl cDNA (equivalent to 50 ng RNA), 0.4 μl of forward and reverse primers (10 μM), 0.2 μl ROX reference dye (50X) and 3 μl distilled H_2_O (water for injection) were placed in Step One Real-Time PCR system (Applied Biosystems, Singapore). Thermal cycles were set as the following program: primary denaturation at 95 Ċ for 30 sec and subsequently 40 cycles of polymerase chain reactions at 95 Ċ for 5 sec and 60 Ċ for 30 sec. All experiments encompassed two biological replicates of each cell line in triplicates. The mean of technical triplicates of each sample was used for statistical analysis. The NTC (no template control) was also incorporated in each run of RT-qPCR.

### Reference genes stability analysis

The distribution of the quantification cycle (Cq) values was firstly analyzed. After that, the stability ranking of the nominee reference genes was computed using GeNorm, NormFinder, and RefFinder algorithms. The geNorm calculates M values in which the higher the M values, the lower the expression stability of the nominees. Besides that, pairwise variation analysis via the geNorm also exhibited the optimum number of reference genes needed for data normalization [6]. Excel-based Normfinder obtained from https://moma.dk/normfinder-software/normfinder-faq also evaluates the overall variation of nominees as well as the variation between subgroups. Similar to geNorm, a lower stability value indicates higher expression stability [31]. Another excel-based algorithm, namely BestKeeper, was downloaded from https://www.gene-quantification.de/bestkeeper.html. BestKeepr ranks reference genes via pairwise correlation analysis and calculation of the standard deviation (SD) and the coefficient of variance (CV), which results in higher stability of genes expression when they have lower variations [32]. RefFinder, which is accessible through https://www.heartcure.com.au/reffinder/?type=reference, calculates comprehensive ranking based on the geometric mean of weights in which the weights are assigned to the nominee ranks calculated by four algorithms comprising geNorm, normFinder, Delta Ct, and BestKeeper [33].

## Results

### Expression status of nominee reference genes in hepatic and breast cancer cell lines

**Fig 1:**
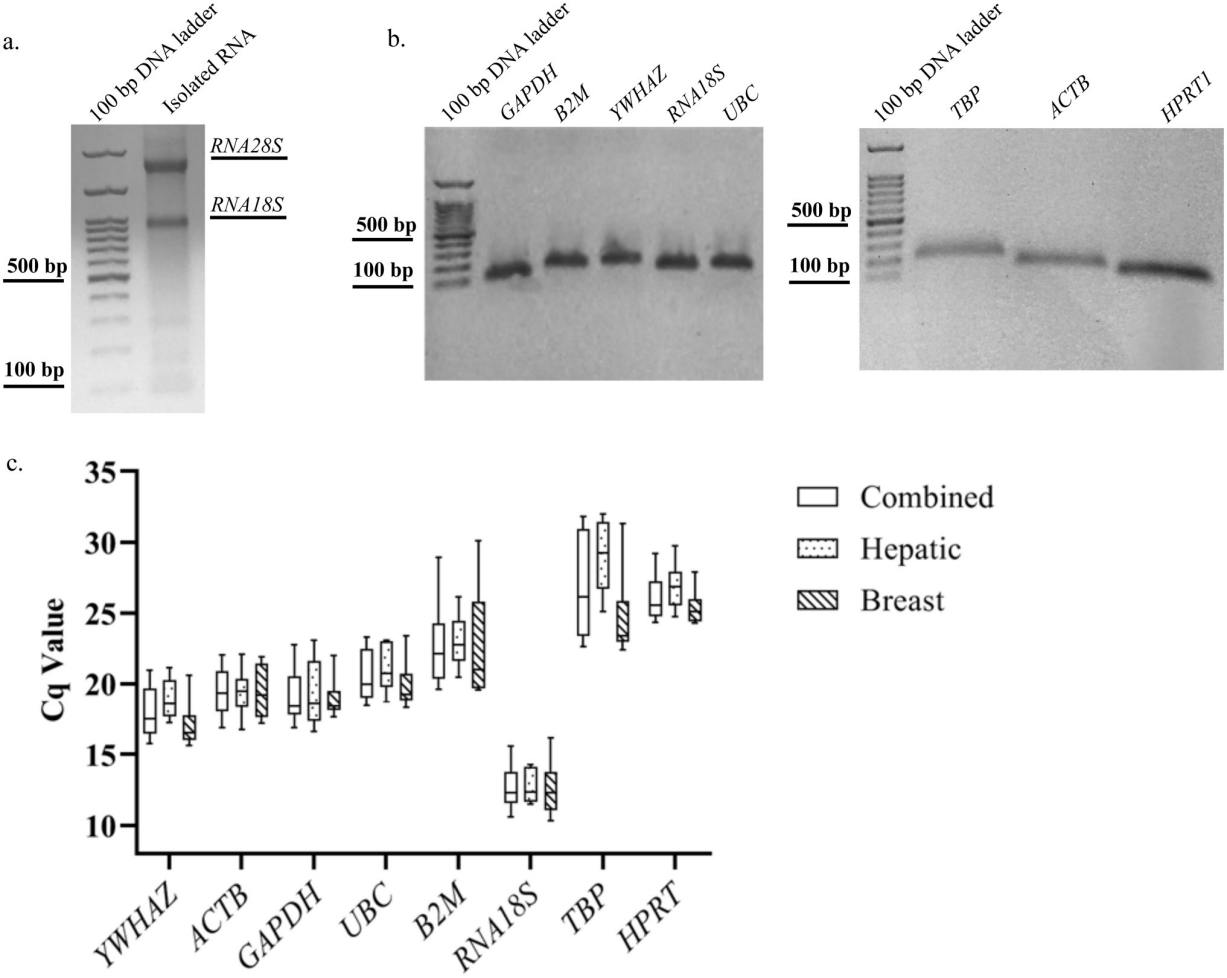
Expression of reference genes and Cq distribution in hepatic and breast cancer cell lines. **A**. Integrity analysis of the isolated RNA derived from SKBR3 cell line. The 2:1 ratio of *RNA28S*/*RNA18S* band intensities on 1% agarose gel confirmed the integrity of the extracted RNA. Similar results were obtained for the other five cancer cell lines. **B**. Specificity analysis of the designed primers. Based on the results obtained from agarose gel electrophoresis, a single band of each gene’s RT-qPCR product at the anticipated size confirmed the specificity of the used primers. The isolated RNA sample (Fig 1A), cDNA template, and the RT-qPCR products (Fig 1B) belonged to the SKBR3 cell line. Full-length gels are presented in S1 Fig. **C**. Cq values distribution of the nominee reference genes in three hepatic cancer cell lines, three breast cancer cell lines, and their combination. The graph is provided as box and whiskers that the latter shows 10–90 percentile.

To display the distribution of Cq values of nominee reference genes, initially, we checked the quality of the extracted RNA samples. As shown in Fig 1A, the integrity of RNA samples was confirmed through the 2:1 ratio of *RNA28S*/*RNA18S* band intensities on 1% agarose gel. Moreover, the range 1.9–2.1 of 260 to 280 nm absorbance ratio showed the purity of the extracted total RNAs. The high PCR efficiency (E), in the range of 1.9–2, calculated via LinregPCR software, indicated the high efficiency of the designed primers in PCR product amplification of the cellular RNA. The PCR efficiency (Mean PCR efficiency ± SD) of all nominee genes is presented in Table 1. A clear band of PCR products on 1% agarose gel electrophoresis (Fig 1B), as well as not seeing primer dimers in the related melt curves, proved the specificity of the designed primers. The efficiency and specificity of these primers except hypoxanthine phosphoribosyltransferase 1 (*HPRT1)*, TATA-box binding protein (*TBP*), and actin beta (*ACTB)* were also studied in exosomal RNA of hepatic and breast cancer cell lines by our team recently [14]. However, here we confirmed their efficiency and specificity in cellular RNA of three hepatic and three breast cancer cell lines.

Cq values distribution analysis (Fig 1C) also showed that in both hepatic and breast cancer cell lines, 18S ribosomal RNA (*RNA18S)* was the most expressed reference gene with the mean Cq ± SD equals to 12.7 ± 1.209 and 12.55 ± 1.994, respectively. While *TBP* (mean Cq ± SD = 29 ± 2.59) and *HPRT1* (mean Cq ± SD = 25.36 ± 1.311) were the least abundant reference genes in hepatic and breast cancer cell lines, respectively. More details in Cq values distribution are presented in Table 2. In Table 2 and all graphs, the combined box or column refers to the statistical analysis of samples of six cancer cell lines in two biological replicates (n = 12). Each of these twelve Cq values is the mean of technical triplicates. Technical triplicates of each sample had differences lower than 0.1 Ct value. Accordingly, hepatic and breast boxes or columns contain Cq values derived from samples of three hepatic cancer cell lines (including two biological replicates for each cell line, n = 6) and three breast cancer cell lines (including two biological replicates for each cell line, n = 6), respectively.

**Table 2:** The Cq values of nominee reference genes in hepatic and breast cancer cell lines.

Genes	Combined			Hepatic			Breast		
	Cq Min	Cq Max	Mean Cq ± SD	Cq Min	Cq Max	Mean Cq ± SD	Cq Min	Cq Max	Mean Cq ± SD
***YWHAZ***	15.63	21.14	17.96 ± 1.834	17.23	21.14	18.90 ± 1.447	15.63	20.6	17.03 ± 1.796
***ACTB***	16.77	22.09	19.43 ±1.704	16.77	22.09	19.42 ± 1.712	17.21	21.89	19.44 ± 1.86
***GAPDH***	16.61	23.08	19.08 ±1.936	16.61	23.08	19.26 ± 2.4	17.67	22	18.91 ± 1.553
***UBC***	18.34	23.38	20.45 ±1.787	18.71	23.09	21.06 ± 1.699	18.34	23.38	19.84 ± 1.801
***B2M***	19.58	30.1	22.82 ± 3.044	20.49	26.15	23 ± 1.93	19.58	30.1	22.63 ± 4.072
***RNA18S***	10.3	16.15	12.63 ± 1.574	11.5	14.29	12.7 ± 1.209	10.3	16.15	12.55 ± 1.994
***TBP***	22.39	32	26.81 ± 3.649	25.09	32	29 ± 2.59	22.39	31.3	24.61 ± 3.321
***HPRT1***	24.29	29.75	26.12 ± 1.647	24.74	29.75	26.89 ± 1.687	24.29	27.89	25.36 ± 1.311

The Mean Cq ± SD values in the combined column refer to the statistical analysis of samples of six cancer cell lines and their biological replicates (n = 12). Accordingly, the hepatic column and breast column contain Mean Cq values derived from samples of three hepatic cancer cell lines (including two biological replicates for each cell line, n = 6) and three breast cancer cell lines (including two biological replicates for each cell line, n = 6), respectively. The Cq Min and Cq Max values in hepatic and breast columns represent the lowest and the highest Cq values across samples of three different hepatic or three different breast cancer cell lines and their biological replicates, respectively. Each of these Cq Min or Cq Max values is itself the mean of technical triplicates.

### Stability status analysis of nominee reference genes employing geNorm excel-based software

**Fig 2:**
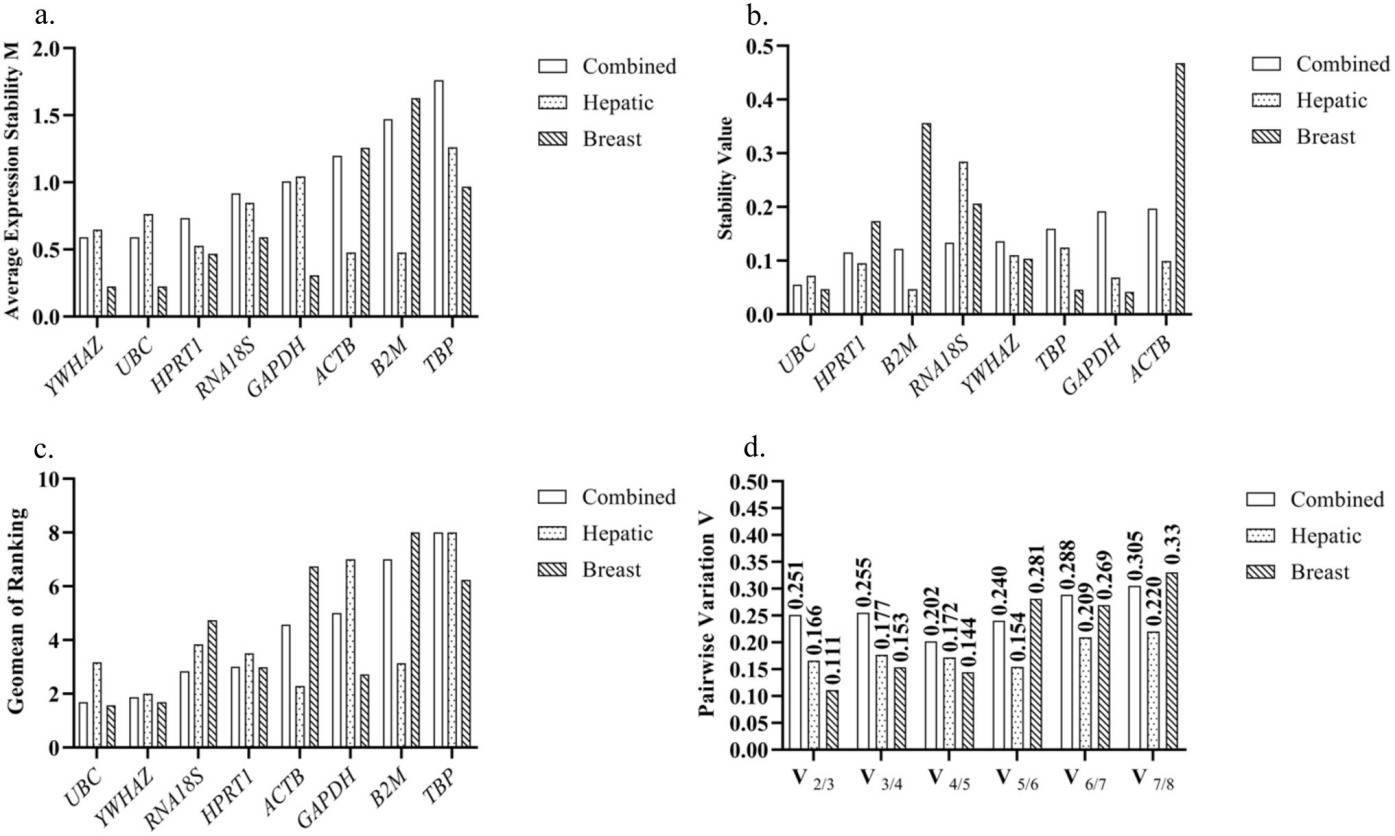
Stability ranking orders of the studied reference genes resulted from A. geNorm, B. NormFinder, and C. RefFinder algorithms in the combined sample besides the hepatic and breast cancer cell lines separately. D. Indication of the optimum number of reference genes in the combined sample besides the breast and hepatic cancer cell lines separately via geNorm algorithm. The combined sample refers to hepatic and breast cancer cells together.

To have appropriate geNorm input data, the mean Cq values of all samples for each reference gene were first transformed to quantities using PCR efficiency (E) according to the geNorm manual. As shown in Fig 2A and Table 3, in HepG2, Huh7, and PLC-PRF5 cell lines, *ACTB* and beta-2-microglobulin (*B2M*) had the lowest M values (0.477). Thereby, *ACTB* and *B2M* are classified as the most stable reference genes equally followed by *HPRT1*, tyrosine 3-monooxygenase/tryptophan 5-monooxygenase activation protein zeta (*YWHAZ*), ubiquitin C (*UBC*), and *RNA18S*, respectively. In MCF7, SKBR3, and MDA-MB-231 cell lines, *YWHAZ* and *UBC* had the least M values (0.222) and the highest stability ranking order. After that, *GAPDH*, *HPRT1*, *RNA18S*, and *TBP* were the most stable ones, respectively. However, *GAPDH* and *TBP* in hepatic cancer cell lines and *ACTB* and *B2M* in breast cancer cell lines have M values > 1, which causes us to consider them as unstable reference genes.

**Table 3:** Stability ranking order of nominee reference genes determined by GeNorm algorithm.

Combined		Hepatic		Breast	
Ranking Order	Average Expression Stability (M)	Ranking Order	Average Expression Stability (M)	Ranking Order	Average Expression Stability (M)
*YWHAZ*	0.59	*ACTB*	0.477	*YWHAZ*	0.222
*UBC*	0.59	*B2M*	0.477	*UBC*	0.222
*HPRT1*	0.735	*HPRT1*	0.526	*GAPDH*	0.307
*RNA18S*	0.918	*YWHAZ*	0.646	*HPRT1*	0.465
*GAPDH*	1.008	*UBC*	0.763	*RNA18S*	0.591
*ACTB*	1.198	*RNA18S*	0.847	*TBP*	0.968
*B2M*	1.472	*GAPDH*	1.044	*ACTB*	1.257
*TBP*	1.760	*TBP*	1.26	*B2M*	1.628

Furthermore, when we analyzed both samples together, geNorm ranked *YWHAZ*, *UBC*, *HPRT1*, and *RNA18S* as the most stable nominees, respectively. Whereas *GAPDH*, *ACTB*, *B2M*, and *TBP* were ranked as the least stable ones.

### Stability status analysis of nominee reference genes employing NormFinder excel-based software

To provide NormFinder input data, the mean Cq values of all samples for each reference gene were transformed to the linear scale expression quantities according to the producer’s instruction. The lowest expression stability value exhibits the most stable reference gene. As shown in Fig 2B and Table 4, in hepatic cancer cell lines, *B2M*, *GAPDH*, *UBC*, and *HPRT1* were the first four stable nominees, respectively, and *RNA18S* was determined as the least stable ones. In breast cancer cell lines, *GAPDH*, *TBP*, and *UBC* were the most stable reference genes almost equally. While similar to geNorm, *ACTB* and *B2M* were the least stable ones in breast cancer cell lines. In the combined condition, *UBC*, *HPRT1*, and *B2M* were the first three stable reference genes, respectively. However, *UBC* and *RNA18S* were ranked as the best combination of two reference genes for data normalization (Stability value = 0.062). Moreover, *ACTB* and *GAPDH* were determined as the least stable ones almost equally.

**Table 4:** Stability ranking order of nominee reference genes determined by NormFinder algorithm.

Combined		Hepatic		Breast	
Ranking Order	Stability Value	Ranking Order	Stability Value	Ranking Order	Stability Value
*UBC*	0.055	*B2M*	0.047	*GAPDH*	0.042
*HPRT1*	0.115	*GAPDH*	0.068	*TBP*	0.046
*B2M*	0.122	*UBC*	0.072	*UBC*	0.047
*RNA18S*	0.133	*HPRT1*	0.095	*YWHAZ*	0.103
*YWHAZ*	0.136	*ACTB*	0.099	*HPRT1*	0.173
*TBP*	0.159	*YWHAZ*	0.11	*RNA18S*	0.206
*GAPDH*	0.192	*TBP*	0.124	*B2M*	0.356
*ACTB*	0.197	*RNA18S*	0.284	*ACTB*	0.468

### Stability status analysis of nominee reference genes employing BestKeeper excel-based software

To rank the reference genes stability, analysis by BestKeepr excel based algorithm was also performed. Our analysis showed that in the combined condition, all genes had a standard deviation ± crossing point (SD ± CP) > 1. While reference genes with SD>1 are considered inconsistent by BestKeeper [32]. In contrast to NormFinder output, BestKeeper analysis showed *RNA18S* as the only reference gene with SD<1 (0.98) in hepatic cancer cell lines. In breast cancer cells, similar to geNorm and NormFinder that ranked *HPRT1* as the first five stable reference genes, BestKeeper also indicated *HPRT1* as the only nominee with SD<1 (0.85). The BestKeeper outputs for hepatic and breast cancer cell lines are presented in Table 5. Moreover, similar to the geNorm output, *TBP*, and *GAPDH* for hepatic cancer cell lines and *B2M* for breast cancer cell lines were sorted as the least stable reference genes.

**Table 5:** Stability ranking order of nominee reference genes determined by BestKeeper algorithm.

Hepatic		Breast	
Ranking Order	SD ± CP	Ranking Order	SD ± CP
*RNA18S*	0.98	*HPRT1*	0.85
*YWHAZ*	1.11	*GAPDH*	1.03
*ACTB*	1.11	*UBC*	1.18
*HPRT1*	1.11	*YWHAZ*	1.19
*UBC*	1.31	*RNA18S*	1.34
*B2M*	1.43	*ACTB*	1.44
*GAPDH*	1.89	*TBP*	2.23
*TBP*	2.05	*B2M*	3.07

### Stability status analysis of nominee reference genes employing RefFinder tool

Finally, we analyzed the overall ranking of all nominees employing the RefFinder online tool. The mean Cq values were entered into the input area of the webpage. The overall ranking orders derived from the RefFinder algorithm are presented in Fig 2C and Table 6. Similar to the geNorm and NormFinder algorithms, *UBC* was graded as the most stable reference genes in the combined condition. Moreover, *B2M* and *TBP* were indicated as the least stable ones, similar to the results of the geNorm algorithm. In hepatic cancer cell lines, the first six stable reference genes were *YWHAZ*, *ACTB*, *B2M*, *UBC*, *HPRT1*, and *RNA18S*. *TBP* and *GAPDH* were also showed the lowest expression stability. Slightly different ranking orders of these genes were displayed by the geNorm algorithm. In breast cancer cell lines, exactly the same ranking order as the geNorm algorithm was reported in which *UBC* and *B2M* were the most and the least stable genes, respectively.

**Table 6:** Stability ranking order of nominee reference genes determined by RefFinder algorithm.

Combined		Hepatic		Breast	
Ranking Order	Geomean of Ranking	Ranking Order	Geomean of Ranking	Ranking Order	Geomean of Ranking
*UBC*	1.68	*YWHAZ*	2	*UBC*	1.57
*YWHAZ*	1.86	*ACTB*	2.28	*YWHAZ*	1.68
*RNA18S*	2.83	*B2M*	3.13	*GAPDH*	2.71
*HPRT1*	3	*UBC*	3.16	*HPRT1*	2.99
*ACTB*	4.56	*HPRT1*	3.5	*RNA18S*	4.73
*GAPDH*	5	*RNA18S*	3.83	*TBP*	6.24
*B2M*	7	*GAPDH*	7	*ACTB*	6.74
*TBP*	8	*TBP*	8	*B2M*	8

### The optimum number of reference genes for reliable normalization of RT-qPCR

Assessing the pairwise variation through the geNorm software was carried out to find the optimum number of reference genes for each sample type. Based on the report of Vandesompele *et al*., 0.15 V value is usually considered as a cut-off point which means that if V_n_/V_n+1_ was lower than 0.15, the addition of another reference gene is not needed for RT-qPCR data normalization [6]. As shown in Fig 2D, V_2_/V_3_ for breast cancer cell lines was 0.111, which pointed out that utilization of the first two stable reference genes is adequate for RT-qPCR data normalization in the three studied breast cancer cell lines. Although 0.15 is usually considered as a cut-off value, the 0.15 value is not a strict cut-off point. Indeed, with the lowest V_n_/V_n+1_ value, the “n” presents the optimum number of reference genes. Accordingly, our results showed that the first five stable reference genes are required for reliable data normalization when studying target gene expression in Huh7, HepG2, and PLC-PRF5 cell lines. Also, V_4_/V_5_ equals 0.202 indicated that the use of at least four reference genes for the correct normalization of RT-qPCR data has been proposed by geNorm when both hepatic and breast cancer cell lines are studied together.

## Discussion

Gene expression analysis using RNA-sequencing; a high throughput technique, and an older DNA hybridization method; microarray, allows studying the expression of a large number or even the whole transcriptome simultaneously, which have received great successes in discovering the pathological pathways of diseases, especially cancer and diagnosis and prognosis biomarkers [34–37]. However, their high cost is limiting for many laboratories [38]. RT-qPCR is a powerful, rapid, inexpensive, highly accessible, always applicable and accurate method in relative mRNA expression analysis, which is often employed for validation of RNA-seq or microarray results [35, 39]. Moreover, it can also be used alone for relative gene expression analysis of an approximately limited number of genes compared with the RNA-seq method [40]. However, the accuracy of its results strongly relies on the selection of stable reference genes to normalize the data [41]. Since it has been shown that different tissues have different gene expression profiles [6], it is important to uncover and introduce stable reference genes in each tissue type. Today, cancer cell lines are widely used and available for researchers as an accessible sample of a specific cancer type [8]. However, some cell lines may not fully reflect the characteristics of the original tissue [42].

Many published research articles have utilized reference genes to normalize RT-qPCR data without analyzing their stability in the specific condition of that experiment and have chosen them only based on their previous usage or their reputation [43]. Therefore, we decided to evaluate the stability of eight well-known reference genes in three breast and three hepatic cancer cell lines using four algorithms, namely geNorm, NormFinder, BestKeeper, and RefFinder.

Our results overally indicated that *GAPDH*, *YWHAZ*, and *UBC* were the most stable reference genes in breast cancer cell lines, including MCF7, SKBR3 and MDA-MB231, and *B2M* and *ACTB* were the least stable ones. Moreover, in hepatic cancer cell lines including Huh7, HepG2, and PLC-PRF5, *ACTB*, *HPRT1*, *UBC*, *B2M*, and *YWHAZ* were among the first six stable genes ranked by geNorm, NormFinder, and RefFinder algorithm. While *TBP* was ranked among the unstable reference genes in the studied three hepatic cancer cell lines.

We have recently reported the expression and stability status of reference genes for exosomal content of the same hepatic cancer cell lines as this study and SKBR3 as breast cancer cell line [14]. Interestingly, the geNorm showed almost similar stability ranking for cellular and exosomal content, in which *B2M* and *YWHAZ* were ranked as two stable reference genes (M value < 1) in Huh7, HepG2, and PLC-PRF5 cell lines. Similarly, *YWHAZ* was also classified by the RefFinder as the most stable reference gene in the cellular and exosomal content of hepatic cancer cell lines. Regarding the combination of hepatic and breast cancer cell lines, the geNorm indicated *UBC* as the most stable reference gene, and the RefFinder ranked *UBC* and *YWHAZ* as two stable ones in both cellular and exosomal content. Although based on two algorithms, the stability ranking of few reference genes in cellular and exosomal content was similar, other algorithms showed various ranking orders, emphasizing the importance of stability analysis for each experimental condition.

It has been stated that *YWHAZ* and *UBC* were ranked as the most stable reference genes by geNorm and NormFinder when the three cell lines, namely MCF7, HCT116, and HepG2, were studied simultaneously. However, the authors did not analyze the cell lines individually [23]. In another study, *YWHAZ*, along with H2A clustered histone 13 (*HIST)*, showed the lowest stability values of geNorm and NormFinder in apoptosis induced-MCF7 cell line, which suggested their usage as RT-qPCR data normalizer in the studied experimental condition. Though this study reported *GAPDH* as the least stable nominee among the other four reference genes including *YWHAZ*, *HIST*, *ACTB*, and heat shock protein family A (Hsp70) member 8 (*HSC70*), more detailed reviewing of the results showed that the stability values (M) derived from the geNorm algorithm were lower than 0.6 for all five reference genes [44]. Moreover, in a study by McNeill *et al*., among eleven nominee reference genes in breast cancer tissue, *GAPDH* was categorized as the fourth stable gene via the geNorm algorithm that was more stable than *HPRT1* and *B2M* [45]. Nevertheless, existing evidence indicates that the expression of *GAPDH* in breast cancer tissues or cell lines would be changed under different pathological conditions or experimental treatments. For example, the association of *GAPDH* expression with the aggressiveness of breast cancer and its upregulation in oestradiol treated MCF7 cells [46] or its downregulation in amino-bisphosphonate treated MCF7 and T47D cell lines [47].

Besides that, analyzing the stability of reference genes in breast cancer tissues by Kilic *et al*. has shown the instability of *ACTB* and *B2M* in which the M values derived from the geNorm algorithm were higher than 1.5 [48]. In a similar study designed by Majidzadeh *et al*., although *ACTB* and transferrin receptor (*TFRC*) had the lowest M values of the geNorm software, even the lowest M value was higher than 1.5, indicating their instability in breast cancer tissues [49]. Our previous study regarding stability analysis of reference genes in *RAB5A*-knocked down Huh7 cells via five different algorithms determined that *ACTB* and *HPRT1* had the highest stability grade among the other six reference genes. While *TBP* showed the opposite stability status [15]. Moreover, another study showed that in interferon-alpha treated Huh7 and HepG2 cells, a combination of *GAPDH* and *HPRT1* had the lowest stability value of the geNorm algorithm. The NormFinder also showed *HPRT1* as the most stable genes. *TBP* was also ranked as the least stable gene using both algorithms [50].

Although here we also ranked reference genes in a combined manner of both breast and hepatic cancer cell lines, due to the Cq variations between different tissues, it is not recommended to use the same genes for both tissue types unless the reference genes stability is analyzed under the specific situation of the experiment.

## Conclusion

According to the obtained results, to normalize RT-qPCR data of MCF7, SKBR3, and MDA-B231 cell lines reliably, it is recommended to use *YWHAZ*, *UBC*, and *GAPDH* as reference genes or further evaluate their expression stability in a specific experiment involving the above three cell lines. Similarly, a panel of five reference genes, namely *ACTB*, *HPRT1*, *UBC*, *YWHAZ*, and *B2M* is also recommended for RT-qPCR data normalization of three hepatic cancer cell lines, including Huh7, HepG2, and PLC-PRF5. Moreover, for the studied cell lines, it is proposed that not to use *ACTB* and *B2M*; and *TBP* for normalizing the breast and hepatic cancer cell lines derived RT-qPCR data, respectively. Since cells experimental conditions like passage number and different treatments can change the stability ranking orders of reference genes [51, 52], considering the stability and instability status of the eight reference genes via four algorithms provided in this study, would initially help to find the most stable RT-qPCR data normalizers of breast and hepatic cancer cell lines.

## Supporting information

S1 Fig Uncropped and unprocessed images of 1% agarose gel electrophoresis with the indicated areas that are presented in Fig 1a and 1b of the article. A. Integrity analysis of the isolated RNA samples derived from SKBR3 cell line. B. Primer specificity analysis of PCR products. The isolated RNA sample (S1A Fig), cDNA template, and the RT-qPCR products (S1B Fig) belonged to the SKBR3 cell line.
(PDF)
