# Supplementary material for: Uncovering the stability status of the reputed reference genes in breast and hepatic cancer cell lines
Source: PLoS One. 2021 Nov 9;16(11):e0259669. doi: 10.1371/journal.pone.0259669 (PMC8577734; doi:10.1371/journal.pone.0259669)
Supplement: S1 Fig — A. Integrity analysis of the isolated RNA samples derived from SKBR3 cell line. B. Primer specificity analysis of PCR products. The isolated RNA sample (S1A Fig), cDNA template, and the RT-qPCR products (S1B Fig) belonged to the SKBR3 cell line. (PDF) [file pone.0259669.s001.pdf]

Supplementary file

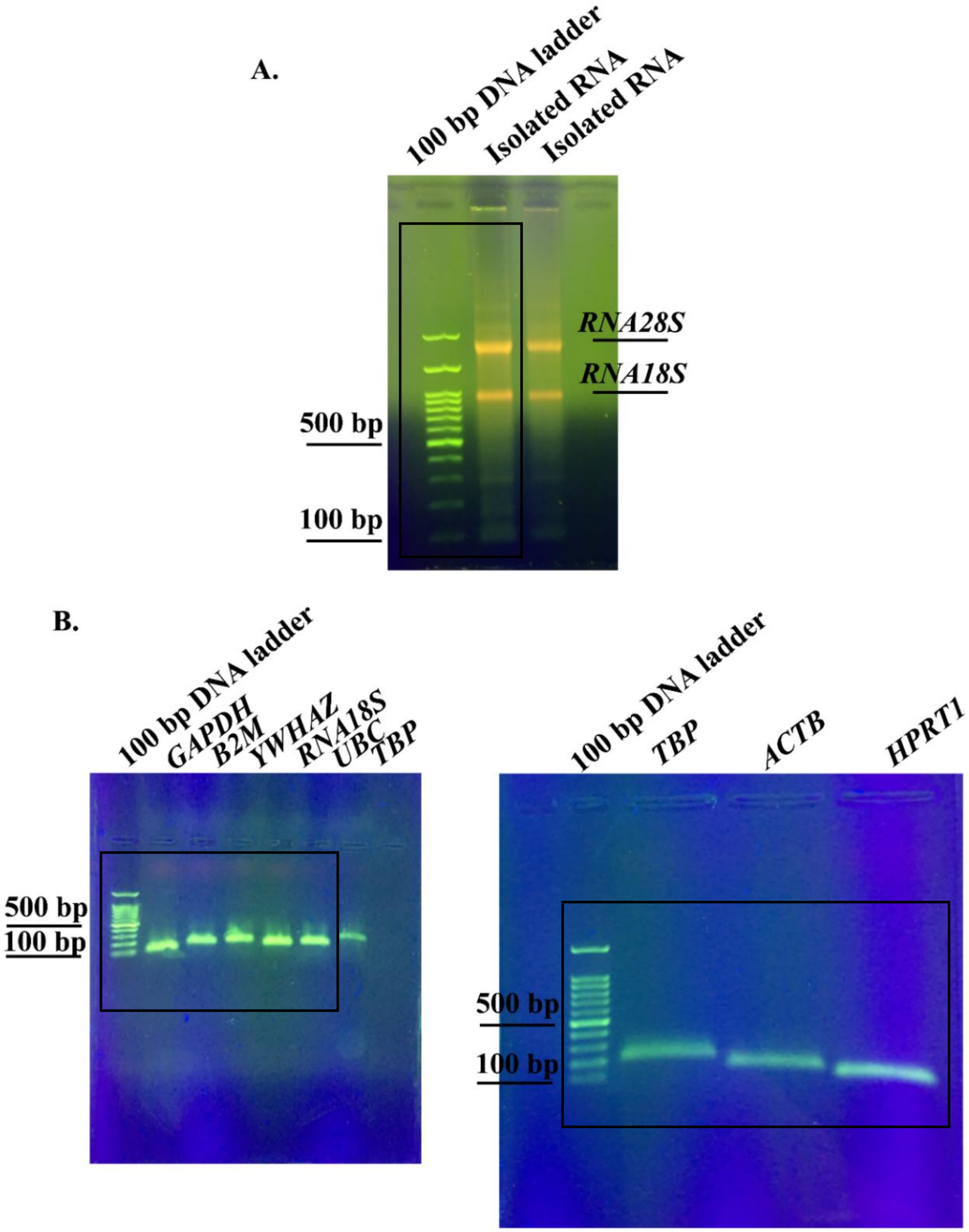

Fig S1. (Related to Fig 1a and b). Uncropped and unprocessed images of 1% agarose gel electrophoresis with the indicated areas that are presented in **Fig 1a and 1b** of the article. A. Integrity analysis of the isolated RNA samples derived from SKBR3 cell line. B. Primer specificity analysis of PCR products. The isolated RNA sample (Fig A), cDNA template, and the RT-qPCR products (Fig B) belonged to the SKBR3 cell line.
